# Suspending the next turn as a form of repair initiation: evidence from Argentine Sign Language

**DOI:** 10.3389/fpsyg.2015.01326

**Published:** 2015-09-15

**Authors:** Elizabeth Manrique, N. J. Enfield

**Affiliations:** ^1^Language and Cognition Department, Max Planck Institute for PsycholinguisticsNijmegen, Netherlands; ^2^Department of Linguistics, The University of SydneySydney, NSW, Australia

**Keywords:** conversation analysis, Argentine Sign Language, visual-gestural modality, “*freeze-look”*, other-initiation of repair, questions, responses

## Abstract

Practices of other-initiated repair deal with problems of hearing or understanding what another person has said in the fast-moving turn-by-turn flow of conversation. As such, other-initiated repair plays a fundamental role in the maintenance of intersubjectivity in social interaction. This study finds and analyses a special type of other-initiated repair that is used in turn-by-turn conversation in a sign language: Argentine Sign Language (Lengua de Señas Argentina or LSA). We describe a type of response termed a “*freeze-look,”* which occurs when a person has just been asked a direct question: instead of answering the question in the next turn position, the person holds still while looking directly at the questioner. In these cases it is clear that the person is aware of having just been addressed and is not otherwise accounting for their delay in responding (e.g., by displaying a “thinking” face or hesitation, etc.). We find that this behavior functions as a way for an addressee to initiate repair by the person who asked the question. The “freeze-look” results in the questioner “re-doing” their action of asking a question, for example by repeating or rephrasing it. Thus, we argue that the “freeze-look” is a practice for other-initiation of repair. In addition, we argue that it is an “off-record” practice, thus contrasting with known on-record practices such as saying “Huh?” or equivalents. The findings aim to contribute to research on human understanding in everyday turn-by-turn conversation by looking at an understudied sign language, with possible implications for our understanding of visual bodily communication in spoken languages as well.

## Introduction

People in interaction use and interpret meaningful hand and facial gestures spontaneously and frequently as part of their efforts to express themselves and to understand others when formulating turns in conversation. In spoken languages, these visible behaviors form an integrated multimodal system with speech, where the visible and audible signs are linked pragmatically, semantically, and temporally (McNeill, 1992; Kendon, 2004; Enfield, 2009). In sign languages, visible behavior bears the entire load: People rely solely on visual-gestural communication when producing linguistic signs and communicative gestures, coordinating multiple bodily resources including manual, facial, and head signs and movements (Klima and Bellugi, 1979; Emmorey, 2001; Sandler and Lillo-Martin, 2006). But no matter what combination of modalities and semiotic resources is used, all language users are faced with the challenge of maintaining mutual understanding in the turn-by-turn flow of conversation (Clark, 1996).

Problems of perception or understanding in conversation occur very often, with other-initiation of repair occurring on average around once every 100 s (Dingemanse et al., in press). To understand how these alerts are handled in real time, we must focus on the basic organizational structure of everyday conversation, namely the sophisticated systems of turn-taking (Sacks et al., 1974; Stivers et al., 2009; Levinson and Torreira, 2015) and sequence organization (Clark, 1996; Schegloff, 2007). When a person asks a question, they are taking a turn at talk of the kind that obliges another person to produce an answer or other relevant response in the next turn. Different responses can be displayed. An addressee can simply answer a question directly if that is possible. But if they do not understand or do not hear the question clearly, they have the option of initiating repair by the questioner, for example by saying in English “*Sorry?,” “What?,” “Huh?,”* or “*Can you repeat that?.”* This is called *other-initiation of repair*, abbreviated as OIR (Schegloff et al., 1977; Dingemanse et al., 2013; Dingemanse and Enfield, 2015).

Research on other-initiation of repair to date has been done almost exclusively on spoken languages, in telephone and face-to-face interaction, with a fairly limited sample of languages beyond English (see Dingemanse and Enfield, 2015; Hayashi et al., 2013 for recent crosslinguistic studies). Despite an explosion of recent research on sign language in linguistics and related fields, there is relatively little research on interactional structures and mechanisms in sign languages, especially where such research focuses on naturally-occurring interaction. Available studies deal with aspects of turn-taking in American Sign Language (Baker, 1977), tactile Sweden Sign Language (Mesch, 2001), Brazilian Sign Language (McCleary and Leite, 2013), and Sign Language of the Netherlands (de Vos et al., 2015), as well as repair practices in American Sign Language (Dively, 1998), and Tactile Australian Sign Language (Willoughby et al., 2014). The present study draws on an extensive corpus of videotaped conversation (both dyadic and multi-party) in a sign language, giving extensive access to spontaneously occurring data on repair practices that rely solely on the visual-gestural modality.

Though repair practices have been traditionally defined as dealing with problems of “speaking, hearing, and understanding” (Schegloff et al., 1977), in sign language these must instead be understood as problems of “*signing, seeing*, and understanding.” Signers use a variety of body articulators in coordinated ways to produce visible linguistic information: these include hand movements, facial expressions, eye gaze, head, and body postures and mouth action signs (Baker, 1977; Baker-Shenk, 1983; Sutton-Spence and Woll, 1999; Boyes-Braem et al., 2001; Liddell, 2003; Sandler and Lillo-Martin, 2006; Vermeerbergen et al., 2007). Other-initiated repair can in principle be produced by any of these articulators or, more commonly, by a combination of them in the repertoire of Argentine Sign Language (LSA) practices (and in other sign languages). Ongoing research on LSA is investigating the full set of types of OIR found in a conversational corpus (Manrique, in press). The “freeze-look” behavior described and analyzed in this article is one of these OIR types.

There are obvious and important differences between the role of the visual modality in spoken vs. signed languages, and it may be expected that these affect the ways in which people encounter and handle problems of perception and understanding. We will distinguish between the *seeing problems* that can lead to other-initiation of repair in sign languages, vs. the *hearing problems* that can occur in spoken languages. Yet there have been recent suggestions of strong commonalities between signed and spoken languages in this domain. Enfield et al. (2013) compare linguistic and conversational mechanisms in relation to problems of understanding in a sample that included LSA and 20 spoken languages across the globe, with results suggesting linguistic and conversational universals in social interaction. LSA signers have the same basic functional options as those described for spoken languages, such as “open” vs. “restricted” formats for other-initiation of repair (Dingemanse and Enfield, 2015; see below for definitions). Another study (Floyd et al., 2014), focusing on the use of “holds” in OIR sequences in LSA and two unrelated spoken languages, Italian and Cha'palaa, also suggests commonalities across signed and spoken languages concerning the function and timing of final-turn holds. In this study visual bodily, including head, face, hands, or torso, or any combination of these components, was compared when initiating repair to another person. This visual bodily behavior is characterized by the maintenance of at least one of these components as strategy of pursuing a resolution of understanding problems. This study has shown that in most of the cases the hold behavior was disengaged only once the person who has initiated repair had heard or seen some or all of the repair solution-turn produced by the person of the trouble source.

If we are going to understand how systems of turn-taking are managed in real time, it is crucial to understand how problems of perceiving or understanding are dealt with on the spot. After all, given the fast pace and constant forward progression of turn-by-turn conversation, if a problem is not fixed immediately then the chance to fix it may quickly be lost. This paper provides a perspective from sign language analysis with the broader aim to gain a better understanding of the general phenomenon of repair as a back-up mechanism for possible threats to the collaborative progress of conversation.

Other initiated-repair occurs necessarily in dialogue, and specifically within the context of conversational turn-taking. A basic OIR sequence has three turn elements (Dingemanse et al., in press). The center or pivotal point in the sequence is the *initiation of repair* (here referred to as T0). T0 points back to the previous turn and identifies it as problematic in some way. This previous turn is termed the *trouble source* of the sequence (referred to as T-1). Usually, T0 explicitly asks that T-1 should be fixed in some way: examples are *Huh?, What?, Who?*. Following T0 is the *repair solution* (or T+1), produced by the person who produced the original trouble source turn. We refer to the producer of the trouble source and repair solution as Person A, and the person who initiates the repair sequence as Person B. If the repair solution by Person A is not sufficient to solve the problem, then Person B might pursue with another initiation of repair asking for more clarification or repetition, thus expanding the sequence. Once B is satisfied with the solution he or she may provide an *uptake turn* (T+2) indicating or at least claiming that he/she has now satisfactorily heard/seen or understood what was said (Schegloff et al., 1977; Clark, 1996).

Our focal point of interest here is the linguistic format of T0, the nuclear turn of the sequence, in which other-initiation of repair is done. T0 turns can display different forms to indicate different problems of perception or understanding in spoken language. Two macro categories of OIR are defined by how they specify the scope of the problem that Person B is targeting in the previous turn. These are “open” and “restricted” categories of OIR (Dingemanse and Enfield, 2015). Open type repair initiators do not specify what the problem is or where it is located in the previous turn produced by Person A: examples of open type repair initiators include *Huh?* and *Pardon?*. These repair initiators point to the entire previous turn as problematic. By contrast, restricted type repair initiators specify what the problem is and where it is located: examples include *Who?* and *They said what?*. These specific types of repair initiators limit the scope of the problem, indicating that the problem is not with the entire previous turn but a part of it.

Previous research on other-initiation of repair has focused on *explicit* or *on-record* ways of initiating repair on other participants' turns. If a speech act is on-record this means that it is non-deniable. For example, if one makes a threat in on-record form (e.g., “If you don't pay up I will hurt your family”), then one would be unable to plausibly deny (say, in court) that it had been a threat. By contrast, a communicative act is done *off*-record “if it is done in such a way that it is not possible to attribute only one clear communicative intention to the act” (Brown and Levinson, 1987, p. 211). An off-record strategy (such as “You should probably pay up. By the way, how is your family? It would terrible if something happened to them”) might be obvious in its communicative intention and yet that intention would be plausibly if not at least technically deniable. Off-record strategies are typically used when people want to avoid possible consequences of being held to account for having performed certain social actions. This is sometimes for legal reasons as in the case of the threat, or perhaps more often it is a way of minimizing the “face-threatening” nature of many types of speech act (Brown and Levinson, 1987). If someone does a communicative act in an off-record way, they are technically leaving it open to the other person to decide how to interpret that act.

In the domain of other-initiation of repair, if a person says “Huh?” or similar known OIR strategy then they are initiating repair in an on-record way. They would be unable to deny that they had intended to momentarily suspend the progress of the conversation in order to resolve a problem of perception or understanding. Here we aim to expand current knowledge of OIR systems by describing a systematic *off*-record practice for initiating repair. This is the “freeze-look,” observed here in LSA: a question is posed, but this question is a source of trouble for the one who is required to provide an answer; rather than providing an answer, the addressee produces a *freeze-look*, meaning that they hold their body and manual articulators still while gazing directly at their interlocutor. In these cases signers continue looking at the questioner without giving any signal that an answer is coming soon. They do not move, and are thus not visibly “gearing up” to respond. We find that the questioner typically treats this practice in the same way as they would treat an open format of other-initiated repair (such as “Huh?”), namely, by repeating or rephrasing the question. We argue that this “freeze-look” behavior is a dedicated but off-record practice for open other-initiation of repair in LSA. The practice allows us to distinguish between on-record OIR and off-record OIR practices in visual-gestural modality in a sign language, and it suggests a distinction in OIR strategies that might be found in other languages, including spoken languages.

## Argentine sign language (LSA)

LSA is used in Argentina, mostly in the city of Buenos Aires, Greater Buenos Aires, Cordoba, and Mendoza. According to the last official report (INDEC^1^) in 2010, there are 289,321 hearing-impaired people in Argentina out of a total population of the country of 41.499 million people. However, there are no official surveys regarding LSA users. LSA is influenced in some ways by contact with Spanish, for example in the common use of Spanish words, either mouthed or fingerspelled. Members of the LSA community vary with respect to their background: a small minority are deaf with deaf parents, most are deaf with hearing parents, others are hearing but have learned to use the language, for example because their parents or other family members are deaf. Beyond the schooling system, deaf clubs and associations provide a context in which LSA is used and learned.

LSA is historically related to Italian Sign Language (Veinberg, 1996). Previous work on the language includes mainly description of the grammar (Massone and Machado, 1992; Massone and Curiel, 1993; Curiel and Massone, 2004), dictionaries (Massone, 1993; Valassina, 1997), and work on deaf bilingual education, interpretation, and other issues (Behares et al., 1990; Veinberg, 1996).

## Data and method

The LSA data used in this study were sampled from recordings of everyday informal dyadic and multi-party interactions made in Deaf clubs and Associations in Buenos Aires, Argentina. LSA users usually meet in these places to interact and share social, educational, sport and political activities. The recordings were filmed without modifying the natural and daily environment of the signers where they normally carry out their activities. The recordings were done as unobtrusively as possible. Participants were not given any instructions or tasks to perform. All participants were native LSA^2^ adult friends. Both men and women took part. The materials were collected with fully informed consent under formal ethics clearance approved by the funding body (European Research Council) and the host institution (Max Planck Society), and also in line with ethical guidelines of the DOBES program (Documentation of Endangered Languages). All the videos were recorded in institutional settings where the relevant authorities authorized the recordings in advance. All participants were informed about the purposes of the research (namely, the study of language use in naturally occurring interactions) and all gave consent before being filmed. Participants signed informed consent statements that provided information about the study, the researchers, and the institutions responsible. They gave permission for the data to be used for research and educational purposes including academic and educational publications. The informed consent forms were written in Spanish and were also translated into LSA by the first author (a certified interpreter in LSA), who collected the data.

The video corpus was collected by the first author between 2010 and 2012. It was filmed using two high definition cameras (Canon HDV). For this study, a sample was taken from the larger corpus by selecting segments of between 10 and 20 min from different recordings to ensure a variety of interactions and participants, totalling 1 h and 50 min of conversation. In this selected part of the corpus 59 signers have participated, between 20 and 80 years old, 35 men and 24 women. Two hundred and thirteen cases were collected to form a set of cases of other-initiation of repair (OIR) for a large-scale comparative research project (see Dingemanse and Enfield, 2015; Manrique, in press). The cases collected were transcribed and translated in collaboration with native signer consultants. From this set, 10% (23 out of the 213) were identified as cases of the “freeze-look” behavior we focus on here. 23 signers, 15 men and 8 women, between 20 and 65 years old have participated in this smaller collection of examples. These “freeze-look” cases were transcribed, glossed, annotated, and translated into English in the transcription software ELAN (Wittenburg et al., 2006). The transcription consisted of sign-by-sign translation into Spanish^3^ following the original sign order, done in collaboration with native LSA consultants. These were annotated using sign language glosses and linguistic notational conventions based on the Johnston (2010) system for sign languages, drawing also on Jefferson's spoken language conversation notation system (Jefferson, 2004, 2015), with some innovations specific to LSA (Manrique, 2011). The examples collected for the OIR collection were translated into English.

### Coding

The basis for identifying and coding the “freeze-look” behavior for this study include formal criteria of the behavior, and distributional criteria in terms of the conversational sequence in which the behavior occurs. The formal criterion of the “freeze-look” action itself is that the body is held still and the gaze is directed straight at the other person. This alone is not enough, though: there is also a distributional criterion for this study, namely that the behavior occurs immediately after a question by the other person in a conversation. The “freeze-look” cases were identified for this study in the context of a larger study of other-initiation of repair (OIR) in LSA and other languages. Subsequent sections provide the details on how the cases were identified and coded.

#### Identification and coding of other-initiated repair (OIR)

A coding system for OIR was created as part of a major comparative project of video-recorded corpora in 12 languages, including LSA (see Dingemanse et al., in press for a detailed description of the coding schema). The design of the coding system was based on observations of conversational data, taking into account extensive prior work on OIR, mainly in spoken English, and enriched with special attention to cross-linguistic diversity and multimodal information.

Sequences of other-initiation of repair in LSA were identified and annotated, using multiple tiers in ELAN to code information about grammatical, pragmatic and sequential properties of each case. Independent tiers were created per participant to annotate grammatical and pragmatic information including independent tiers for signers' right and left hands, and for non-manual markers including: eyebrows (raised, together), eyes (wide open, squint, closed), eye gaze, wrinkled nose, mouth gestures, mouthing, head movements, and upper-body movements. Each example was identified with a unique ID, and the three core turns of each sequence were distinguished: (1) trouble source (e.g., A: *Have you seen John?* = T-1), (2) initiation of repair (e.g., B: *Who?* = T0), and (3) solution turn (e.g., A: *John.* = T+1), along with an “uptake” or sequence-closing turn if relevant (e.g., B: *Ah, no, I haven't seen him today.* = T+2). Table 1 shows the distribution and frequency of types of OIR cases in the collection (Manrique, in press). In LSA, restricted type repair initiators are nearly twice as frequent as the open type.

**Table 1:** **Frequency of the types of repair initiators in the LSA corpus (Manrique, in press)**.

**Type**		**Subtype**	**Frequency (n/213)**	**Proportion (%)**
Explicit (on-record)	Open	Non-manual^4^	51	23
		Question-word (*What?*)	13	6
		Formulaic	0	0
	Restricted	Content q-word (asking for specification)	15	6
		Repetition (asking for confirmation)	56	26
		Offer (asking for clarification)	52	24
		Alternative question	3	1
Implicit (off-record)	Open	“Freeze-look” response	23	10

#### Coding and transcription of “freeze-look” cases

“Freeze-look” cases were coded for numerous features, including timing aspects and formal aspects. Three measures of timing of “freeze-look” cases were coded on independent tiers in the annotation software. These timing measures were as follows:

- the length of the entire sequence (T-1, T0, and T+1);
- the duration of the “freeze-look” (see Section Timing of “Freeze-looks” below);
- the time between the end of the question (T-1) and the beginning of the (near) repeat of the question (T+1), both produced by *A* (see Section Timing of “Freeze-looks” below).

Formal coding of the “freeze-look” cases included the information that we provide in the data examples below, presented using between a minimum of one line and a maximum of five lines. Two lines are distinguished for non-manual markers (NMM), the first one for head movements (e.g., head-down) and the second one for facial movements (e.g., ET “eyebrows together”). The line below NMM information (see schema below) indicates the extension and alignment of NMM (above the line) in relation to manual makers (below the line) that are often produced in overlap. In general, one line is used for manual sign glosses (mainly lexical information, illustrated in line 3 below) giving single-word translations into English in capital letters. In some examples it is also relevant to include more information to indicate distinctive use of the separate hands. In these cases, one line is used for the right hand and another one for the left hand. Mouthing is also used frequently in OIR practices and it is indicated by a separate line (see line 4) after the manual glosses when it is relevant. The last line corresponds to the free English translation in italics. Here is an example, illustrating the distinct lines for representation of each of the formal aspects that we coded.

**Figure:**
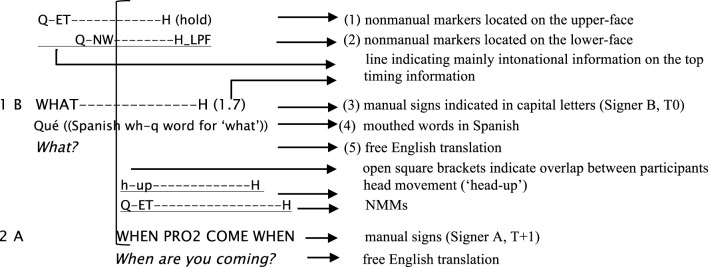


A *large open bracket* indicates when overlapping turns are produced between participants. At the end of line (3), the timing information of the duration of a sign is indicated between parentheses in seconds (1.7). In line (4), double parentheses contain additional comments from the transcriber (see Supplementary Material for a full description of conventions used in the examples in this article). Besides the transcription of the cases described above, a summary of every example is also provided for easier access to the data, including only the main OIR sequence (T-1, T0, and T+1) in free English translation.

## Results

### Question-answer sequences

This study focuses on question-answer sequences in unscripted sign language interaction. Question-answer sequences are one type of *adjacency pair* (Schegloff, 1968; Schegloff and Sacks, 1973; Schegloff, 2007). In an adjacency pair, an initial move by Person A creates a normative requirement for Person B to produce a response, where that response is expected to be of a particular kind. For example, in a question-answer adjacency pair, the first *pair part* (e.g., “What time is it?”) puts the other person in a position where they are obliged to respond appropriately. The preferred response to a question is an *answer* (e.g., “9 o'clock”), but the normative obligation to deal with the question can also be handled by a *non-answer response* that is still relevant to the question (e.g., “Sorry, I don't have a watch”). Both answers and non-answer responses are adequate as second-pair parts to questions. There are, however, more vague or ambiguous types of things one might do immediately after a question. For example, one could stay silent and not move. This could of course be taken as a complete lack of response, if for example Person B did not realize that Person A was talking to them at all. But it could also be taken as a specific way for Person B to provide a non-answer response, not just a failure to respond but a way of signaling that one is not going to respond. This is the possibility we explore in subsequent sections.

Before proceeding, we briefly describe how questions are formed in LSA. Signers in LSA mark questions with non-manual markers (Veinberg, 1993) as also is often the case in other sign languages (Baker and Padden, 1978; Baker-Shenk, 1983; Sandler et al., 2011). The use and the timing of non-manuals are coordinated and linguistically constrained to the manual sign(s) with which they co-occur (e.g., Baker-Shenk, 1983; Pfau and Quer, 2010). The main non-manual markers for questions in LSA are *eyebrows together* for WH-questions (“What?,” “Who?” etc.) and *eyebrows raised* for yes/no questions. These eyebrow positions can be combined with head upward or downward movements and/or upper body leaning forward. Another important characteristic of questions in LSA is the presence of eye gaze directed from the person who asks the question to the addressee of the question. It occurs during and after the question has been produced. At the end of the question, the questioner usually then momentarily suspends or “holds” at least one of the elements that compose the question. These can be manual signs (e.g., “*What?,” “Who?,”* etc.), or non-manual components such as facial actions that indicate that a question has been produced.

#### Fitted responses to questions

When Person A asks Person B a question, Person A ideally expects an answer: this would “fit” best as a response. This is illustrated in the next example. In Extract 1 Signer A asks a question about Signer B's children (line 1) and Signer B immediately provides a fitted answer in the following turn (line 2).

**Figure:**
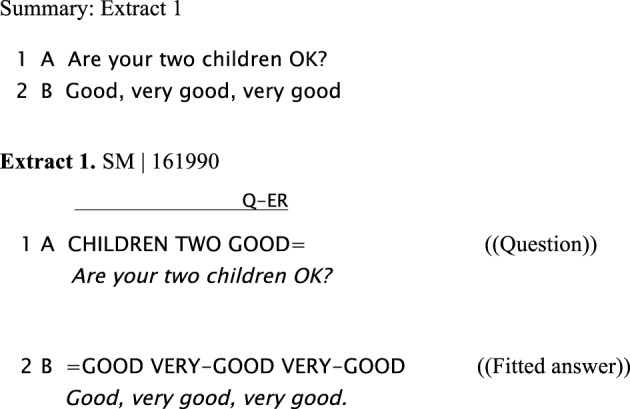


**Figure:**
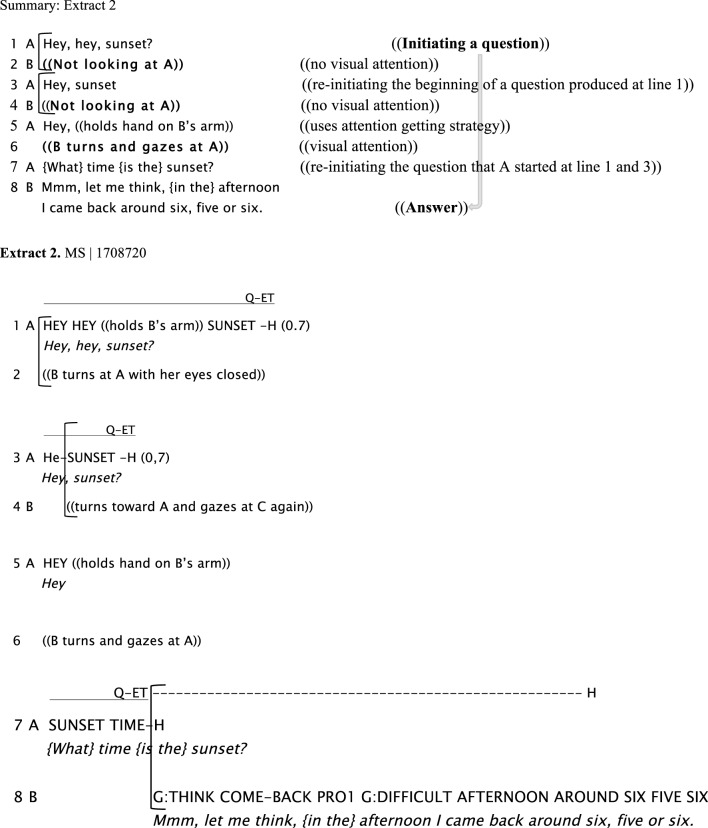


#### Non-fitted response

There are numerous ways in which someone might produce a *non-fitted response* to a question; i.e., something other than an answer. In this section we discuss three types of non-fitted response—non-attendance, word search, and on-record repair—before giving closer attention to a fourth type of non-fitted response, which is the focus of this study.

##### Non-response due to non-attendance

One way of producing something other than a fitted response is not to respond at all. In sign language this can happen if the addressee was not looking at the other person when the question was asked, or if they were interrupted or distracted by someone or something else when the question was asked. In these cases, there is an obvious account for why no response is given: it is clear in the situation that the question was not properly attended to and could not have been perceived or understood. In this situation, the questioner needs to secure the addressee's attention before redoing the question.

The next example shows numerous strategies to get another signer's attention when they are clearly not perceiving what is being signed to them (cf. Baker, 1977). These include directing the eye gaze, tapping the addressee and holding the hands up waiting for the addressee's attention. In the example given below, Signer A starts asking B a question (line 1), but at that moment Signer B is signing and looking at another person, C. Signer A tries again by holding Signer B's arm to get her attention, but B continues signing to C. Then, Signer A maintains the last manual sign she has produced still while looking at B and waiting for her attention. In lines 3 and 4, A re-initiates the question when B (line 4) turns her head toward A. However, A has her eyes closed and looks back to C again. In line 5, A tries again to get B's attention by holding B's arm, B looks at A and A repeats the question (“*Sunset?”*) adding the sign “*time?”* to finish the question “*What time is the sunset (there)?”* This refers to the sunset in a different region in Argentina (Perito Moreno Glacier in the South). In line 8, B answers A's question after several attempts from A. In this example it is clear that the lack of response from B to A's initial attempts to ask her question is due to non-attendance and failure to perceive what was being signed.

Extract 3 shows a similar example in a dyadic interaction between two friends. Although one of the participants (B) is signing to A in this example, he is not maintaining eye contact all the time, but shifting eye gaze, closing his eyes, looking at his hands while occasionally monitoring his addressee. In line 1, when Signer A asks Signer B a question, Signer B is not looking at A. Then, Signer A maintains both hands in signing position, pointing at Signer B (“*PRO2”*), waiting for his attention. In line 3, once B opens his eyes and looks at A, then A repeats the question. B recognizes A is asking a question, drops his last sign and answers A (“*NO, I haven't sent it to the office”*), followed by a clarification, “*I sent it, but I haven't read it.”*

**Figure:**
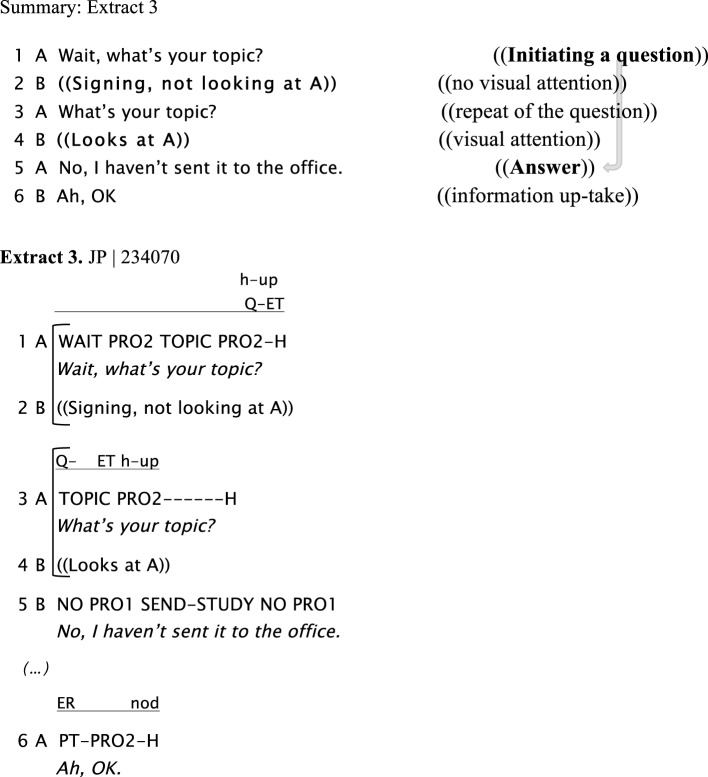


Visual contact and feedback play different roles in signed and spoken language conversation (see Baker, 1977). Constant visual feedback and mutual monitoring between parties in sign language conversation is indispensible to successful communication. Signers generally maintain more focused interactions and minimize multi-tasking activities that would divert visual attention from the interaction. Signers provide constant feedback and monitoring using manual and nonmanual attention-getting strategies (Baker, 1977). More research is needed to determine how different signed and spoken language everyday conversation are in this regard.

##### Non-fitted response with signs of “word search”

A second way of giving a non-fitted response to a question is to give an explicit signal that the response is delayed due to inability to find the words one is looking for in formulating an answer. Word-searching displays are common type of non-fitted response in everyday interaction. They indicate that the addressee is working on the answer and that the answer is delayed. Speakers use different vocal and gestural strategies to indicate they are working on the answer such as: cutoffs, fillers (“*um,” “uh,”* etc.) (Levelt, 1983; Clark and Fox Tree, 2002), and break of eye contact (often then looking upwards) (Goodwin and Goodwin, 1986). Signers use similar gestural visual strategies to indicate problems in delivering or remembering a specific reference, including shifting eye gaze, closing eyes, “thinking” gestures, or rubbing fingers.

Extract 4 shows multiple word searching strategies by both participants after a question is asked. These are: breaking eye contact, squinted eyes (line 4); giving an *ad-hoc* description instead of name (“short hair”), holding hand/s up, and closing eyes (line 5) when trying to retrieve a name using fingerspelling (line 8).

**Figure:**
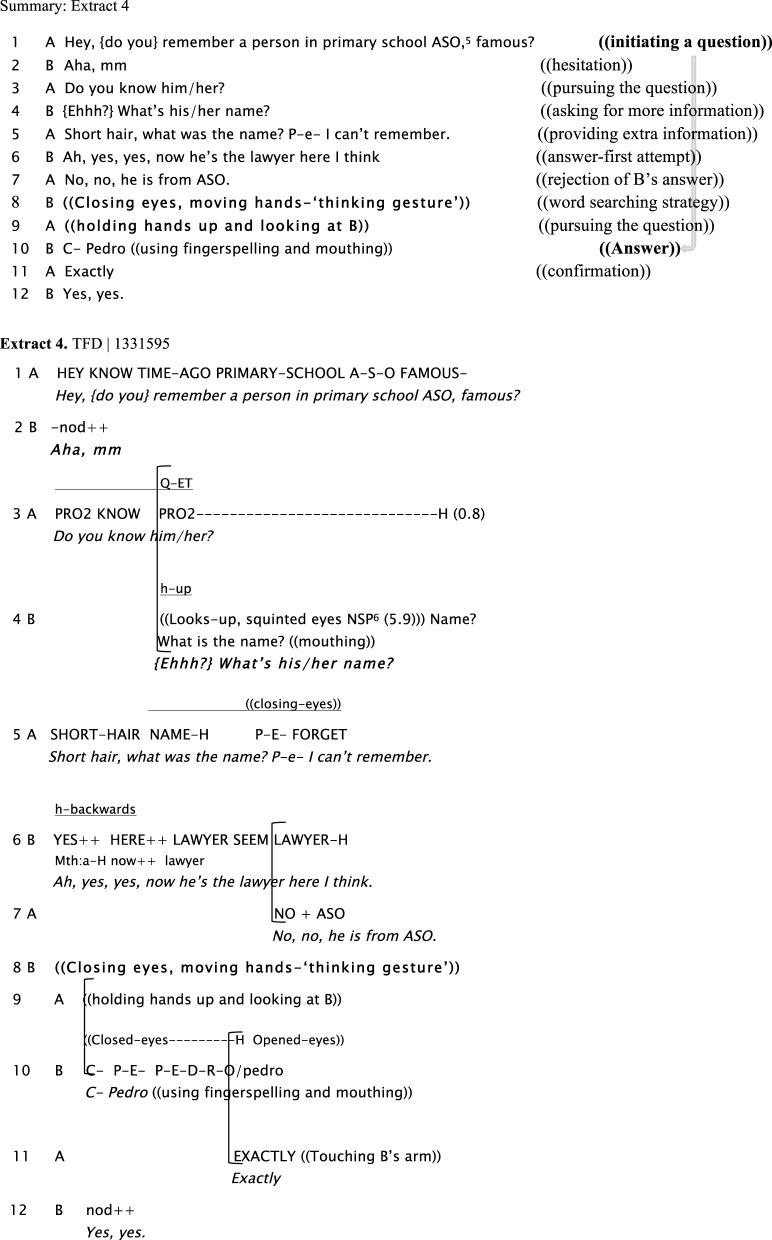


In examples like this one, a signer's observable “thinking” behavior is a way of overtly accounting for the failure to provide an answer to a question. As it also indicates that the signer has indeed understood the question, it does not elicit a repetition of the question.

##### Non-fitted response: on-record repair

A type of non-fitted response that is always possible is an explicit, on-record other-initiation of repair (Schegloff et al., 1977; Schegloff, 1982; Hayashi et al., 2013; Dingemanse and Enfield, 2015). If one has not heard or understood a question, it is always possible to ask for repetition or clarification of the question rather than attempt to answer it. Other-initiation of repair is a way of dealing with online problems of hearing and understanding during interaction so as to maintain and secure mutual understanding, alignment, and affiliation. It is, however, dispreferred, as it halts the progress of talk during a conversation, derailing it momentarily (Stivers and Robinson, 2006).

**Figure 1:**
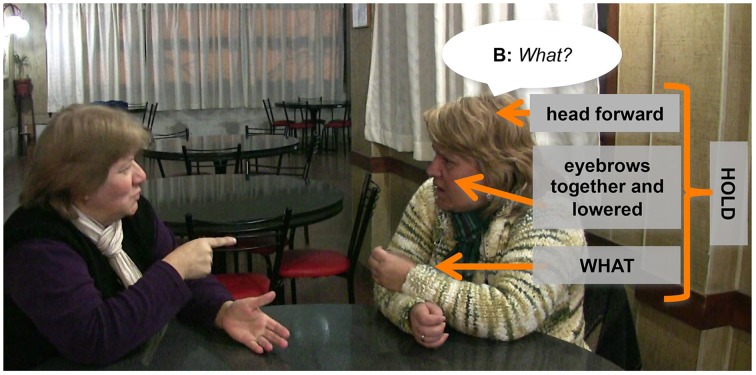
**“*What?,”* Signer B, on the right, initiates an open type of repair on A's prior turn in line 2, producing a manual sign for WH-q word *(“WHAT”*) and nonmanual components (bringing her eyebrows together and leaning forward)**.

Extract 5 shows an explicit initiation of a repair sequence after a question as illustrated in Figure 1 by Signer B. It is done using an “open” format (“What?”) in line 2. There is also a “restricted” type of repair initiation (“Inside?”) by Signer A, in line 5.

**Figure:**
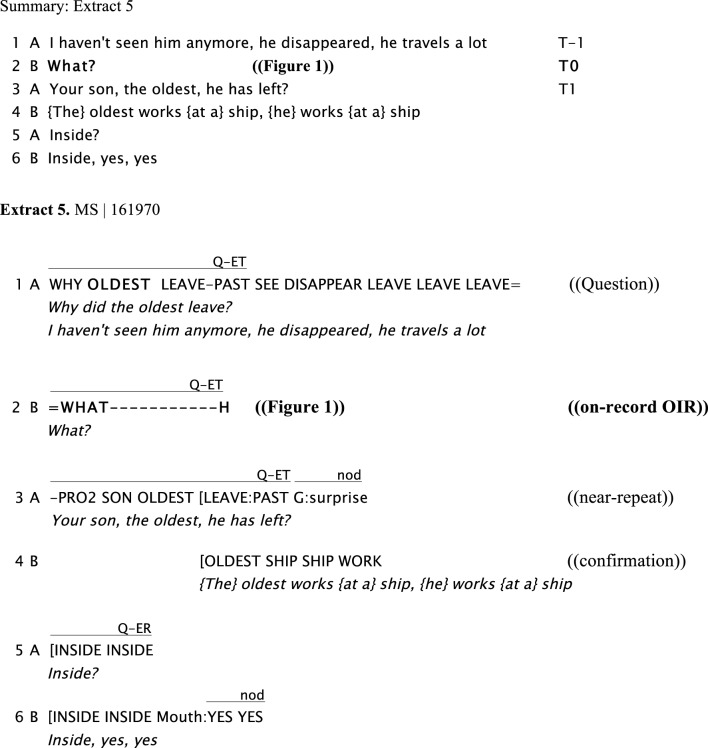


In this example, Signer B displays an explicit initiation of repair using both manual signs (WH-question word “*WHAT”*) and nonmanuals (eyebrows together and leaning forward). Signer B holds these until Signer A solves the problem by near-repeating the trouble source (i.e., the question). In the solution turn, Signer B makes the implicit question more explicit, and more specific (by clarifying the person referent, “*Your son, the-oldest”*). Another initiation of repair in this example is done using a restricted format (line 5; for the terminology “restricted” vs. “open,” see Section Introduction, above, and Dingemanse and Enfield, 2015). It is produced with a combination of manual markers (the hand sign for “INSIDE”) and nonmanual markers of yes/no questioning (raised eyebrows and head moving downwards).

## Freeze-look: a notable absence of response

We now turn to the type of non-fitted response that we refer to as a *freeze-look*. We argue that this type of response is a non-official or off-record way of initiating repair. In a collection of cases of other-initiated repair in LSA (Manrique, in press), the “freeze-look” practice makes up around 10% of all cases. The “freeze-look,” which effectively prompts a questioner to re-do their question, is performed by an addressee by holding their hands and body in position and looking directly at the questioner at a time when it is expected that they should be now responding to the question. This suspended or frozen body posture is maintained until the signer of the trouble source redoes the question (e.g., by repeating or rephrasing), or until the person producing the “freeze-look” upgrades by initiating an on-record other-initiation of repair (see below).

**Figure 2:**
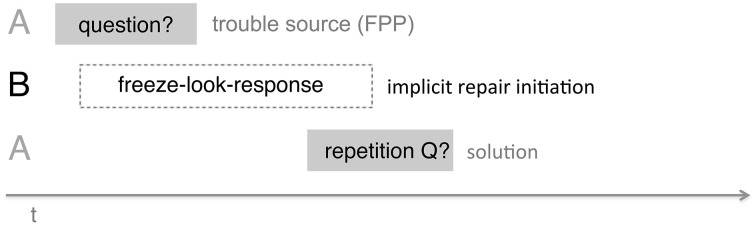
**The typical “freeze-look” response sequence, including the question produced by A, as trouble source and first pair part (FPP), the “freeze-look” as a noticeable absence of response turn and the (near) repeat of the question as solution turn provided by the person who earlier produced the trouble source**.

The definitive characteristics of the “freeze-look” are the following:

- At the relevant moment, the addressee of a question (Signer B) is normatively required to produce a relevant response (an answer to a question, or something related).
- The addressee looks directly at the Signer of the question (Signer A).
- The addressee temporarily holds their entire body posture in a still or “frozen” position.
- It is clear that the addressee has seen that they were just addressed by A; and they are not otherwise signaling any difficulty in responding.
- Signer A then redoes the question (e.g., by repeating or rephrasing).

(See diagram in Figure 2 for a representation of the “freeze-look” response sequence).

We argue that the “freeze-look” is an implicit or off-record practice for initiating repair. Other ways of initiating repair such as asking “What?” are on-record because they use symbolic means to explicitly state that there is a problem of perception or understanding and that this problem now needs to be fixed; the speaker is “officially” committing to their intention to momentarily suspend the progress of the interaction, in spite of possible negative or “face-threatening” effects of this (Brown and Levinson, 1987). By contrast, the “freeze-look” is off-record because it does not explicitly encode the intention to initiate repair, just as “It's cold in here” does not explicitly encode the intention to get somebody to shut the window. Nevertheless, as we argue below, the “freeze-look” is highly effective as an other-initiator of repair, but it still technically leaves the interpretation open, thus making a display of giving the recipient some freedom to decide how to interpret the utterance (Sifianou, 1999).

**Figure 3:**
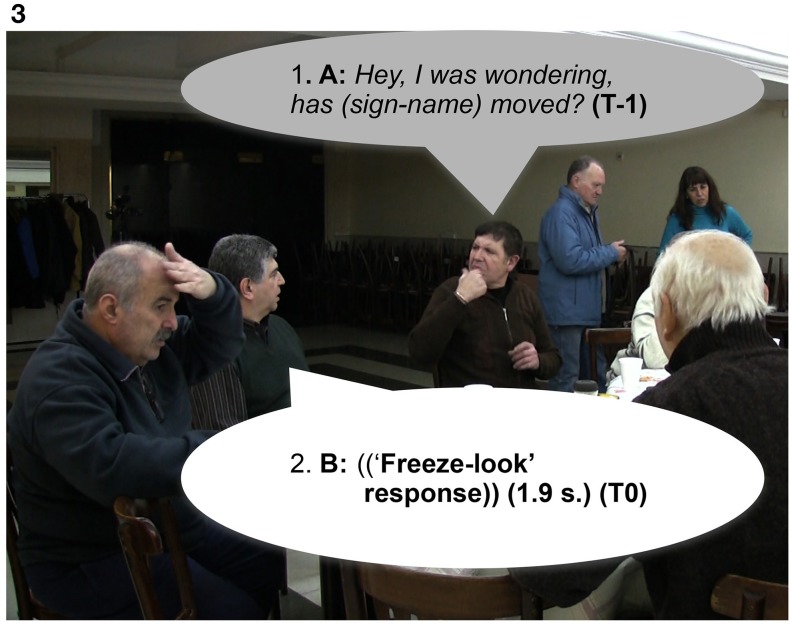
**“*Hey, I was wondering, has (sign-name) moved?,”* Signer A, sitting on the right, asks a question to Signer B, sitting on the left (line1)**. Signer B produces a “freeze-look” response instead of a fitted answer (line 2) that last for 1.6 s.

We now present examples of the “freeze-look” phenomenon. In Extract 6, Extract 7, Extract 8, and Extract 9, Signer B produces a “freeze-look” after Signer A has asked them a question. Signer B suspends her/his signing body posture, maintaining it still from the beginning of Signer A's question until near the end of the re-doing of the question as depicted in Figure 3. The key point we wish to make here is that in all these cases Signer A treats B's “freeze-look” behavior in the same way as they would treat an explicit open format of other-initiation of repair, namely by immediately re-doing the question (with or without some adjustment). In all these cases, once the question is re-done, B can then produce a fitted response.

**Figure:**
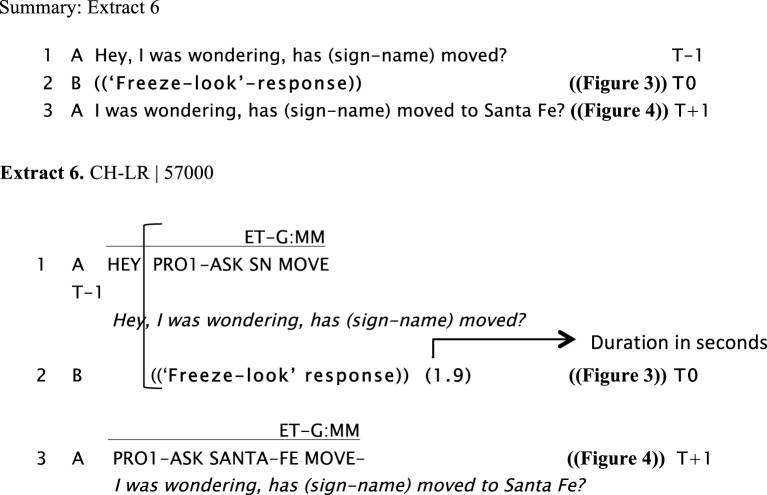


**Figure:**
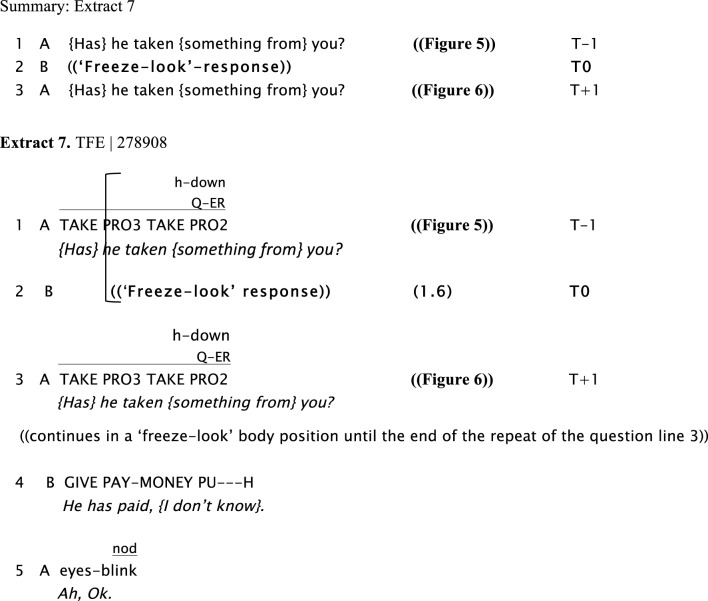


**Figure:**
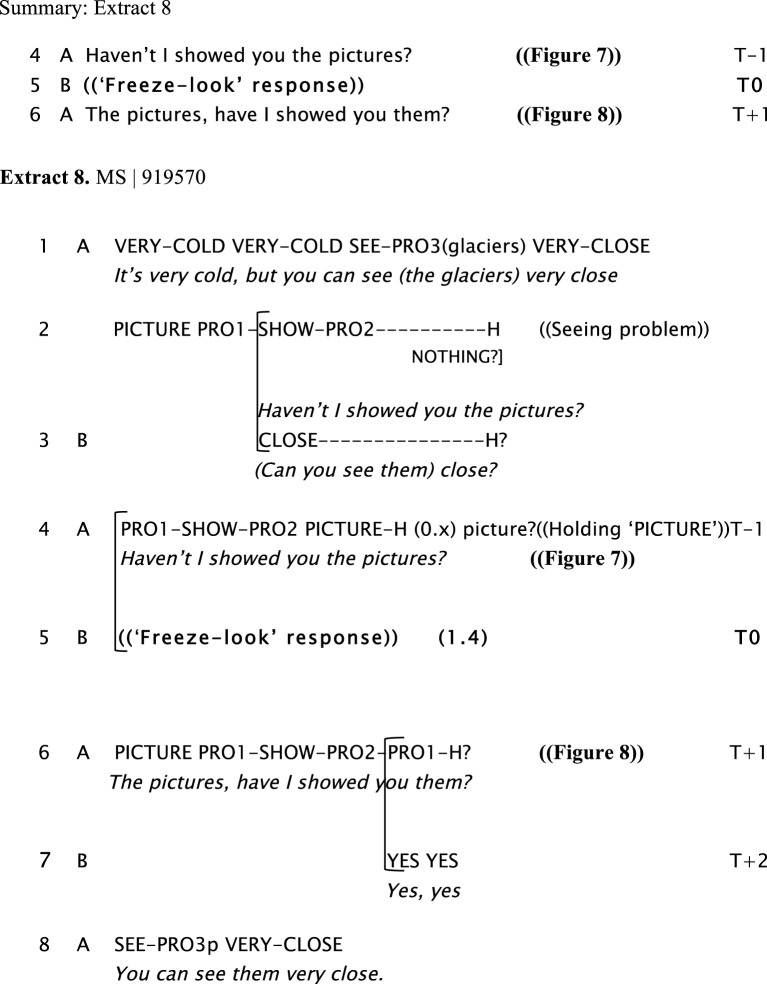


**Figure:**
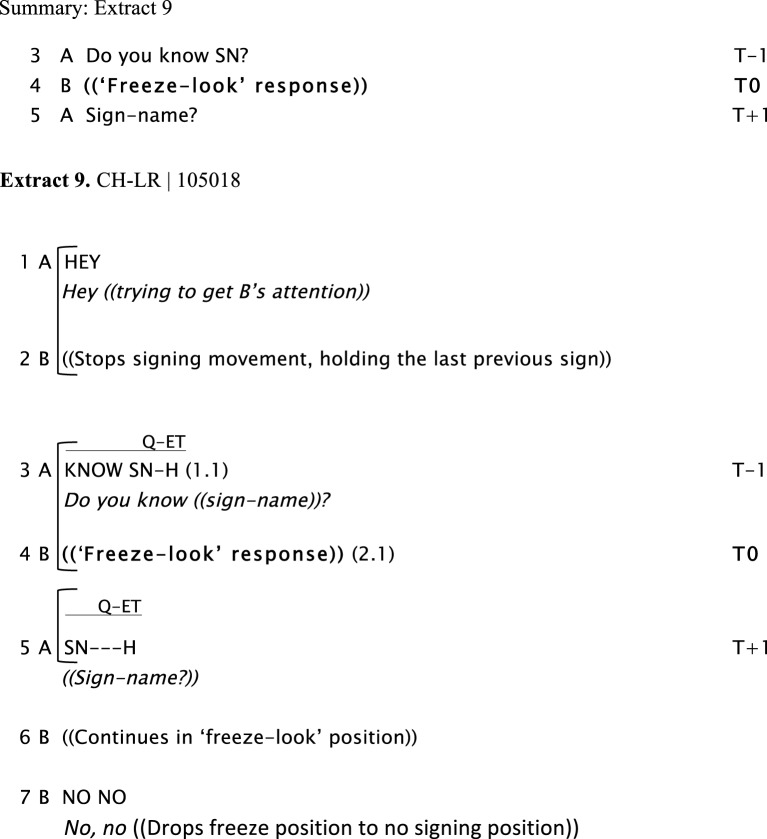


**Figure 4:**
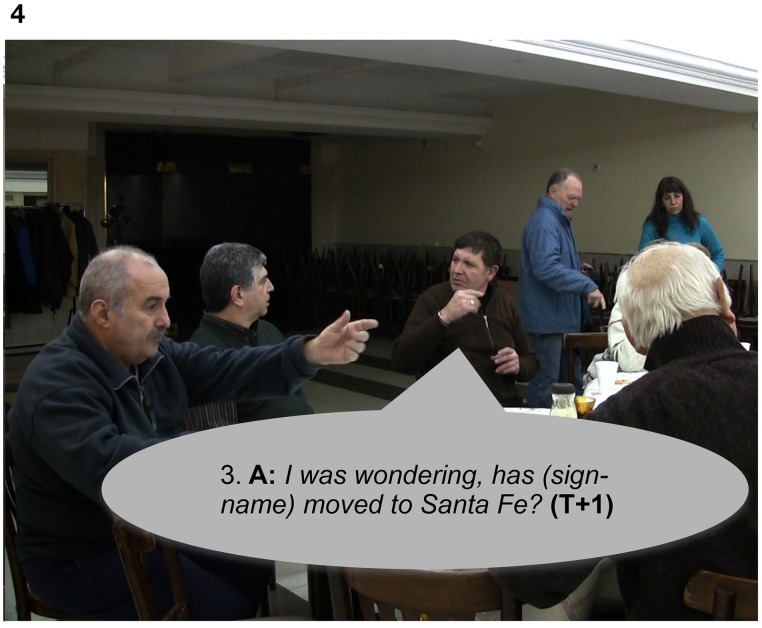
**“*I was wondering, has {sign-name} moved to Santa Fe?,”* Signer A, repeats the question (line 3) produced in line 1 by modifying the order of the utterance and adding more information by specifying a place name**.

In Extract 6, Signer A responds to Signer B's “freeze-look” with a slightly modified repetition of their question, changing the order of the utterance followed by holding his palms up at the end “*eh?,”* and adding more information by specifying a place name (“*Santa-Fe,”* a province in Argentina) (see Figure 4).

**Figure 5:**
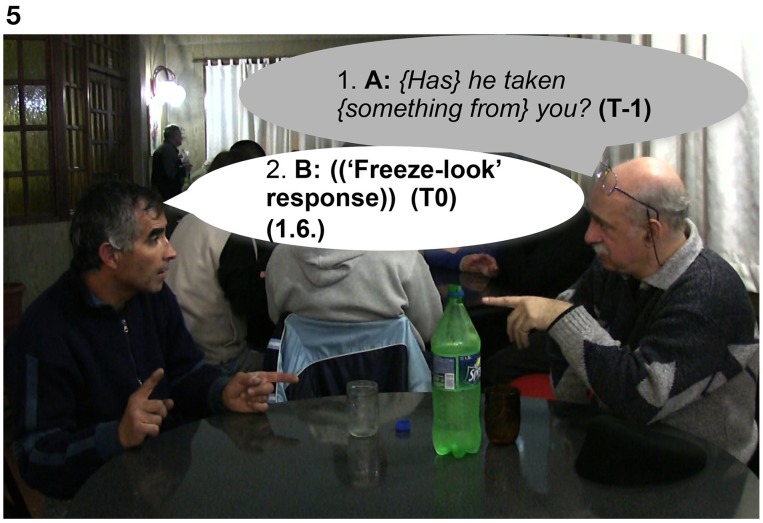
**“*{Has} he taken {something from} you?*,” Signer A, sitting on the right, asks B a question (line 1)**. Then, Signer B, sitting on the left, displays a “freeze-look” response for 1.6 s (line 2).

**Figure 6:**
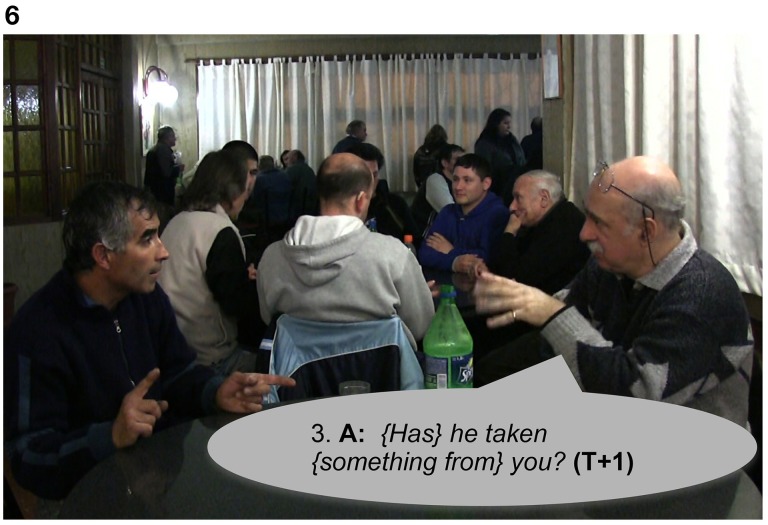
**“*{Has} he taken {something from} you?*,” Signer A, sitting on the right, repeats the question to B (line 3)**. In parallel, Signer B, sitting on the left, continues with the same “freeze-look” until Signer A finishes the repetition of the question (line 3) and then answers the question.

In Extract 7, Signer B produces a “freeze-look” response to A's question, holding still both manual and nonmanual signs for 1.6 s from the beginning of the question until the end of the re-doing of the question represented in Figures 5, 6. Signer A responds to Signer B's “freeze-look” in the same way they would respond to an on-record OIR strategy, namely by immediately offering a repeat of the question.

**Figure 7:**
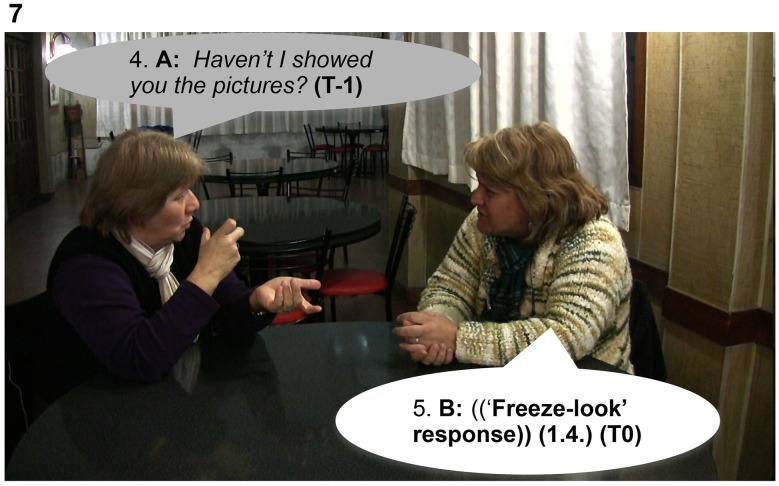
**“*Haven't I showed you the pictures?,”* Signer A asks a question to Signer B, sitting on the right**. Signer B suspends her body position producing a “freeze-look.”

**Figure 8:**
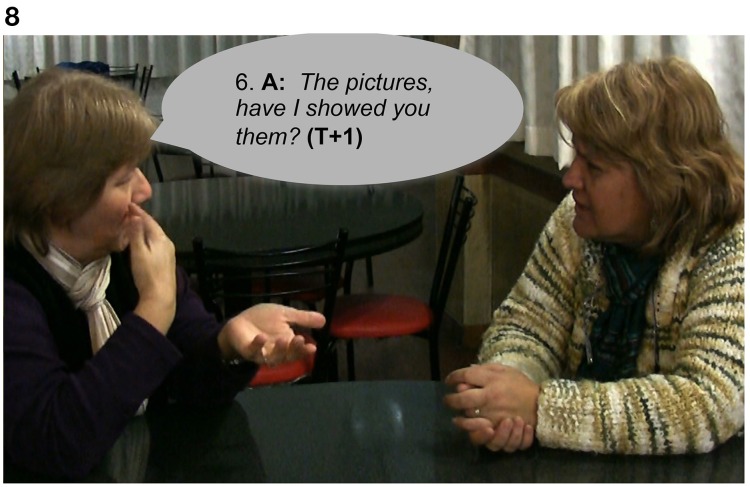
**“*The pictures, have I showed you them?,”* Signer A, sitting on the left, repeats the question and Signer B continues maintaining the “freeze-look” until toward the end of the repetition of the question, when she provides a fitted answer to A**.

In Extract 8, two friends are chatting about vacations in the Perito Moreno Glacier in the South of Argentina. Signer A has vacationed there and Signer B is planning to visit. There is a seeing problem produced by an overlap: both participants are signing at the same time. B's “freeze-look” occurs when Signer A asks the question again: Signer B stops signing, maintaining her nonmanual configuration illustrated in Figure 7. The eventual response from Signer A is a re-doing of the question in line 6: a partial repetition, with a change in the order of signs in the utterance shown in Figure 8. Signer B then produces a fitted answer (in line 7) as the repeated question is coming to an end.

Extract 9 shows a similar case, beginning with an attentional problem: Signer B is not looking at Signer A, and Signer A has to secure B's attention in order to proceed. Once Signer B's visual attention is on Signer A, Signer A then asks B about another person using a sign name (SN), in line 3. From the beginning of A's question in Line 3, Signer B produces a “freeze-look,” looking directly at Signer A and holding still his signing position. Then in Line 5, Signer A repeats the sign name of the person he has asked about. Note that in this case, B's “freeze-look” is held for some time after the end of Signer A's repeated question, and is released only when Signer B begins providing a fitted response.

The sequences in Extract 8 and Extract 9 illustrate the kinds of seeing problems that are common in sign language interaction, and they show that these problems can occur in the run-up to a “freeze-look” sequence. The two examples have a similar structure: Person A asks Person B a question, but B is not attending and fails to respond; Person A then secures B's attention before repeating the question; Person B produces a “freeze-look” response; and finally Person A repeats the question and a fitted answer can be given, thus closing the sequence and allowing the conversation to move forward. These cases help us see a distinction between non-response due to absence of attention (not seeing that one had been asked a question at all) and the open signal of non-response that we term the “freeze-look.” The key difference is revealed in how the non-response is treated by person A. If the non-response is simply due to B's lack of attention, then A will then secure the required attention in some way. If the non-response is in the form of a “freeze-look” from B, then B will repeat the question. Because B is looking directly at A when they produce a “freeze-look,” then the problem cannot be one of attention or perception; instead, because B is studiously not responding, the implication is that they cannot respond, and this will most likely be because they have not clearly comprehended what was just asked. The simple solution is for Signer A to repeat the question: precisely the response that they would have produced had Signer B asked “What did you say?.”

### Pursuit cases: from implicit to explicit OIR

If we are correct in claiming that the “freeze-look” is an *off-record* way to do other initiation of repair, this implies that it is at the “weak” end of the scale of repair strategies (Schegloff et al., 1977, p. 369). This would lead to the following prediction: If a “freeze-look” response to a question does not elicit a repetition or clarification of that question, the person who produced the “freeze-look” can then upgrade to a more explicit or on-record initiation of repair. This prediction is borne out in the LSA corpus. Almost 50% of the “freeze-look” action cases (11 out of 23) are upgraded to an explicit on-record OIR (while the opposite ordering is not observed). In most of the observed cases, a “freeze-look” is upgraded to an open format of other-initiation of repair (such as *What?*), but it may also be upgraded to a restricted format (such as *Who? Where?)*. We now look at some examples.

In Extract 10 Signer B's first response (in Line 2) is a “freeze-look,” but the second version of the question produced by Signer A in response (in Line 3) does not appear to be adequate. Rather than giving an answer to the question, Signer B instead upgrades to an explicit way of initiating repair (i.e., a head tilt that can be translated as “Huh?”). The problem is eventually solved, with Signer B able to answer the question in line 6.

**Figure:**
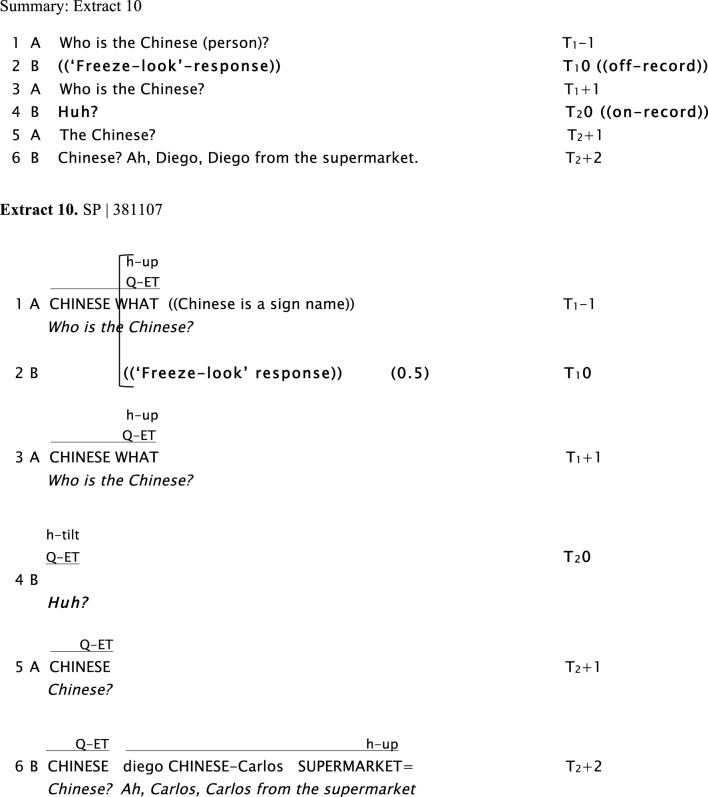


In Extract 11, Signers A and B are chatting about B's son, who is working on a cruise. Signer A asks B if there are many tourists traveling on the cruise (line 1). After a “freeze-look” from Signer B, A repeats the question, with changes to the word order. At this point, rather than answering the question, in line 4 Signer B initiates repair more explicitly, with a sign that combines puckering of the lips and leaning forward of the head (also roughly translatable as “Huh?”), along with mouthing of the Spanish word *Como?*. Finally, in line 5, Signer A solves the sequence by partially repeating the question “*Tourism?,”* adding mouthing to the partial repetition.

**Figure:**
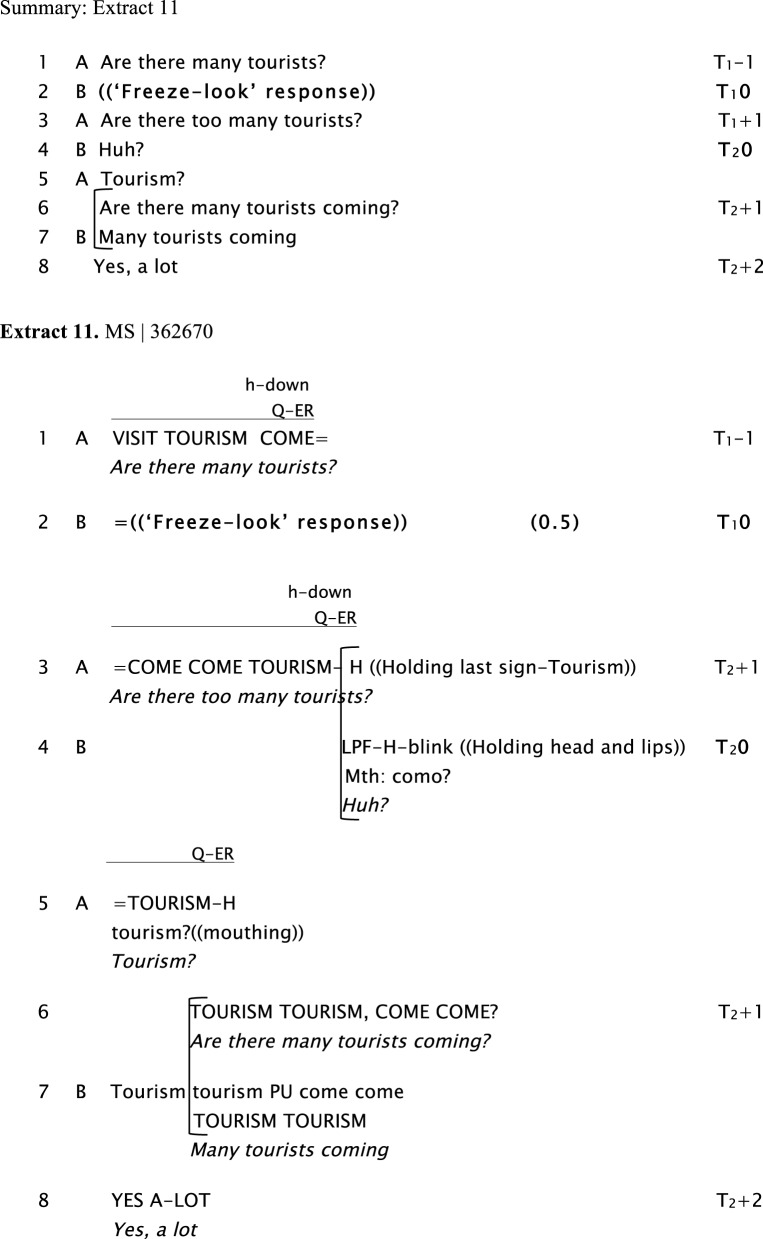


**Figure 9:**
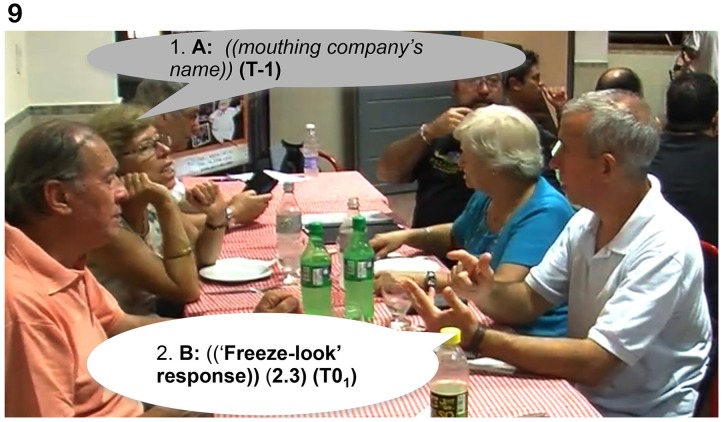
***((“Company's name”))*, Signer A, the woman sitting on the left side of the table, asks a question to Signer B, the man in the white t-shirt sitting on the right side of the table**. At the end of the question, Signer B produces a “freeze-look” (lines 1 and 2) for 2.3 s.

**Figure 10:**
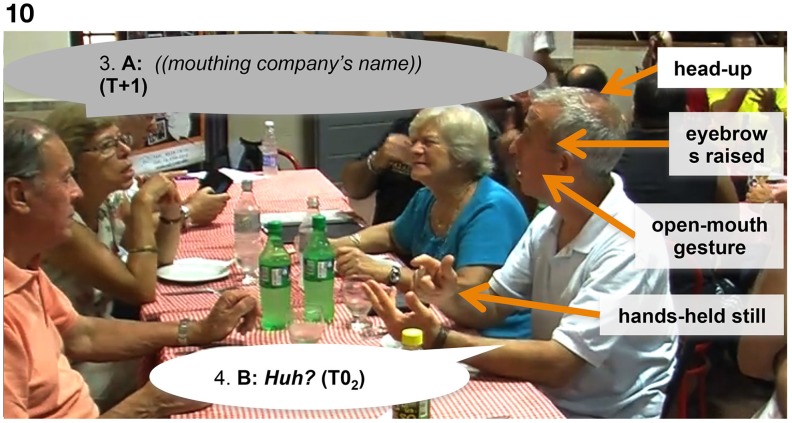
**“*Huh?*,” Signer B, after Signer A repeats the question, initiates a more explicit type of OIR using several NMMs**. He produces an open-mouth gesture (resembling “huh?”), raises his eyebrows and moves his head upwards, while holding the manual signs produced in the previous turn (see Figure 8) to explicitly initiate repair.

In Extract 12, Signer A asks a question using only mouthing (in line 1), in response to which Signer B produces a “freeze-look” response as shown in Figure 9. Signer A does a repeat of the question, again using mouthing. Signer B's response is now a more explicit type of OIR using several NMMs. He produces an open-mouth gesture (resembling the interjection “*huh?”)*, raises his eyebrows and moves his head upwards (see Figure 10), while holding the manual signs produced in the previous turn (se) to explicitly initiate repair. Then, Signer A uses fingerspelling to solve the problem in line 5. Mouthing is a common cause of understanding problems in LSA that is often fixed by using fingerspelling instead (Manrique, in press).

**Figure:**
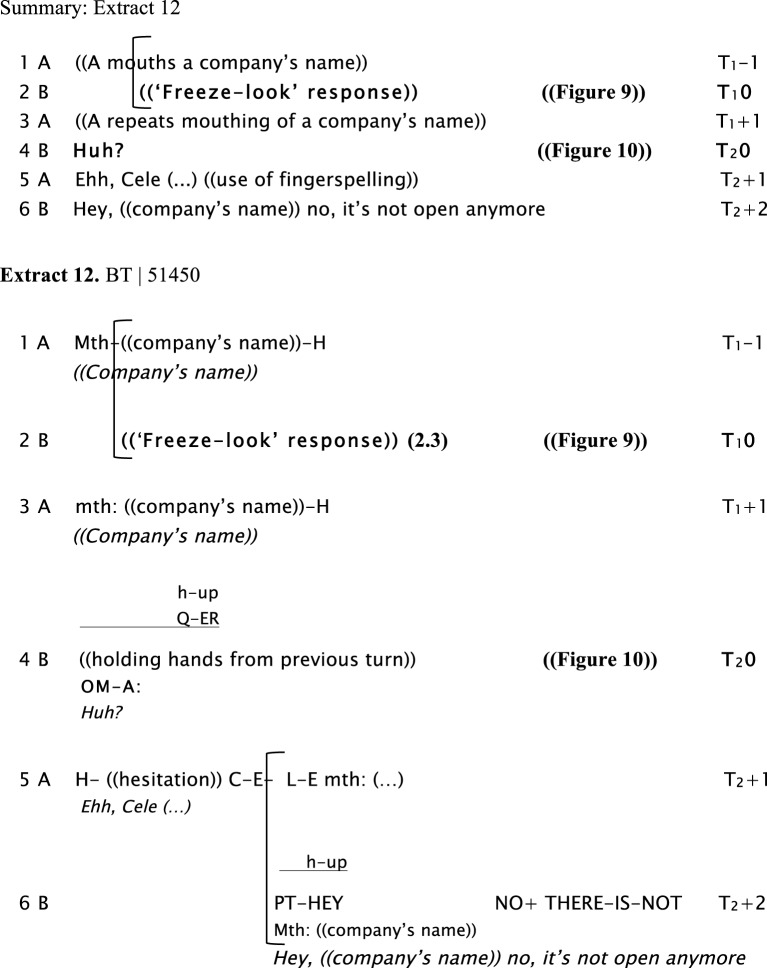


### Timing of “freeze-looks”

**Figure 11:**
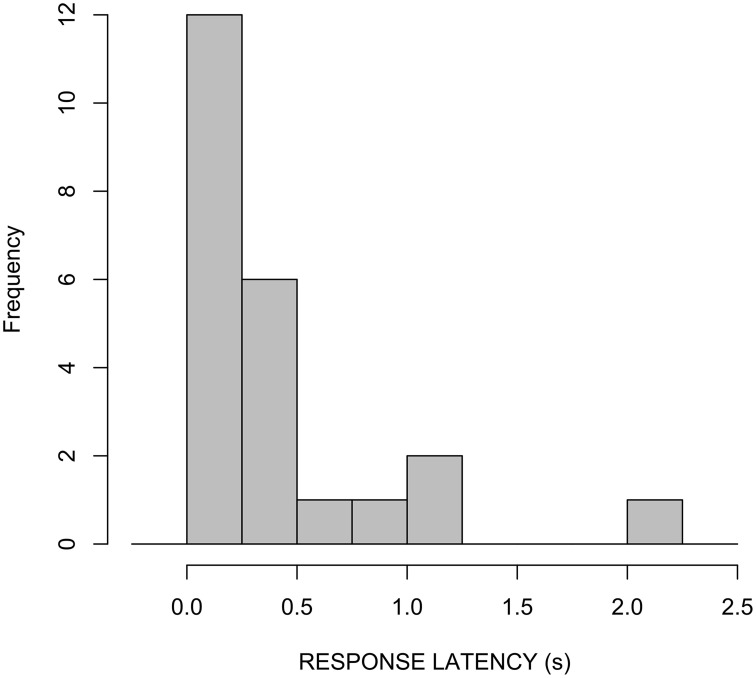
**Response latencies from onset of B's “freeze-look” to beginning of A's repair turn**.

Our study focuses on the function of the “freeze-look” in a specific context (immediately after a question) and identifies a specific function in that context (it elicits a “repair” of the question in the form of a repeat or near-repeat). In addition to measuring the effects of the “freeze-look” by examining the responses it elicits, we also measured aspects of the “freeze-look's” timing. A first measure to note here is the response latency, i.e., the time between the end of the trouble source by Person A (with B producing a “freeze-look”) and the beginning of their “repair” or (near-) repeat of the original question. See Figure 11.

This shows that the “freeze-look” has a rapid effect in interaction: when a person finishes their question and finds that they are faced by their addressee (still) producing a “freeze-look,” then they will quickly follow up with a repeat or near-repeat of the question.

**Figure 12:**
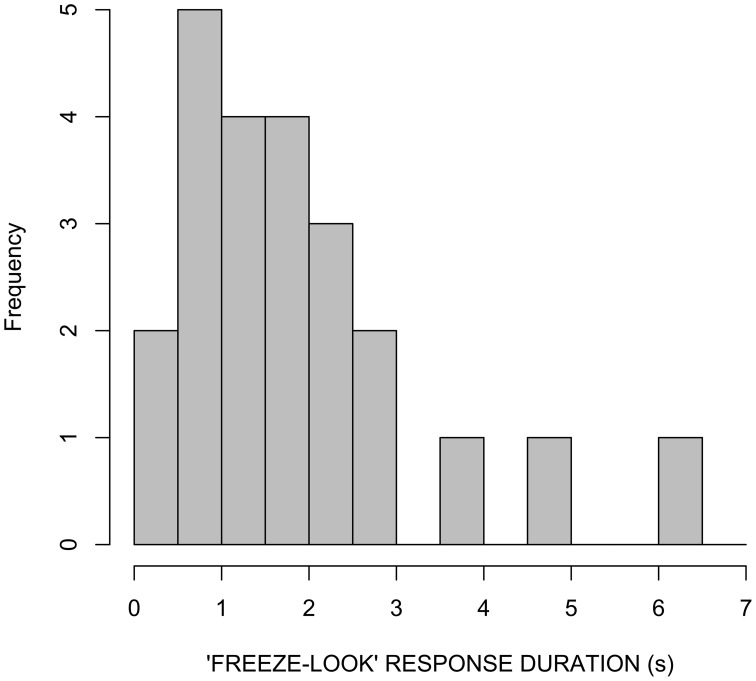
**“Freeze-look” durations in LSA data set**.

A second timing measure to note is the absolute duration of “freeze-look” behaviors. In our LSA data, there is a range in duration from 0.3 to 6.3 s, with 69 percent between 0.5 and 3 s. See Figure 12.

This suggests that “freeze-looks” can continue well past the onset of the “repair” that they elicit, in T+1. This is indeed the case: They tend to be “released” not at the moment at which the subsequent repair turn begins, but rather at the moment at which Person B is ready to produce their next utterance. This will either be when Person B upgrades to a “stronger” OIR because the repair was inadequate for some reason (which happens about 50% of the time), or it will be when Person B produces an “uptake” or similar turn that signals subsequent repair turn was a satisfactorily resolution of the problem (see Floyd et al., 2014 for description of this timing pattern in a three-language comparison, involving LSA and two spoken languages; the form of the “freeze-look” and “hold” in spoken languages is similar to sign language, as it involves the same manual and facial articulators, being used for co-speech gesture).

## Conclusion

**Figure 13:**
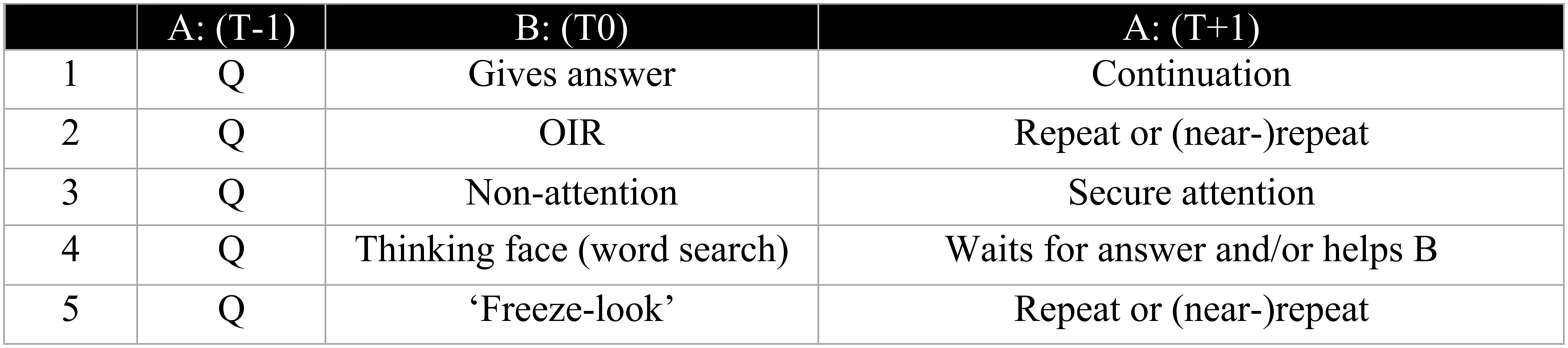
**Possible patterns of response and counter-response after a question, showing functional similarities between “freeze-look” and on-record OIR strategies**.

The evidence we have presented from LSA shows that the “freeze-look” behavior—the act of keeping the whole body in a still position while looking directly at the person who has just asked a question—functions as an open-class other-initiator of repair (OIR), and additionally that it is “off-record” and somewhat weak in nature. Our claim that a “freeze-look” is a kind of OIR is supported by the fact that it gives rise to the same functional outcome as other known types of OIR: namely, it leads to a “re-doing” of the first utterance (e.g., a repeat or a reformulation). Figure 13 summarizes the possible patterns of response and counter-response after a question that have been reviewed in this study, showing explicitly the functional identity or similarity of the “freeze-look” and other available OIR strategies.

**Figure 14:**
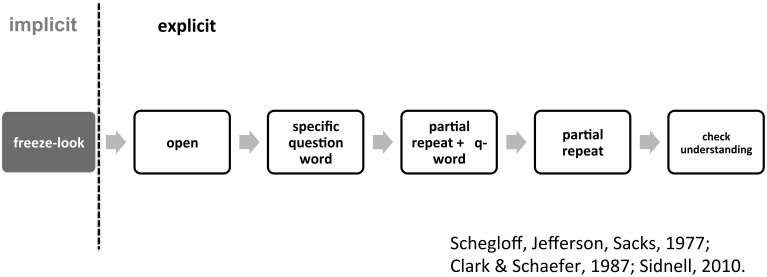
**Types of other initiation of repair laid out on a continuum from “weak” to “strong” (after Schegloff et al., 1977; Sidnell, 2010) with the “freeze-look” behavior placed at the extreme “weak” end of types of conversational repair**.

Our claim that the “freeze-look” is an off-record strategy of OIR is firstly based on its intrinsic semiotic properties: it does not use symbolic resources (i.e., conventional signs such as lexical items that have semantic entailments) to explicitly encode that there is a problem of understanding; instead, it uses non-symbolic resources (i.e., indexical signals of the kind that occur in animal communication; on these semiotic distinctions see Enfield, 2013: Chapter 4). Secondly, its status as off-record is consistent with the fact that it appears to be “weaker” than other available OIR options (just as an indirect request is “weaker” than a direct request). One sense in which it is weak is that it only seems to succeed half of the time it is used: in 50% percent of cases, a “freeze-look” is followed up by a stronger other-initiation or repair. The common upgrading of a “freeze-look” to an explicit OIR (such as “What?”) shows that it occupies a position in a “paradigm” of alternative types of OIR. This was shown in the cases presented in Section Freeze-look: A Notable Absence of Response, above. In each of those cases, Signer B initially produces a “freeze-look” response to Signer A's question, leading to a re-doing of that question; however, the solution appears to be inadequate, and Signer B then upgrades with a stronger, on-record open OIR, indicating that Signer A's first re-doing of the question did not solve the problem. This ordering of Person B's chosen strategies for OIR in these sequences provides evidence in favor of the argument that the “freeze-look” is a weak type, which sometimes needs to be upgraded or strengthened. We propose that this can be captured by placing the “freeze-look” at the extreme “weak” end of a continuum of types of conversational repair (from cf. Schegloff et al., 1977; Sidnell, 2010). Schegloff et al. (1977) rank the OIR formats in in terms of their “strength” in identifying the trouble source of the OIR sequence. “Open-class” repair initiators (Drew, 1997) have been placed on the “weakest” end, as they leave open the identification of the trouble source; often, the entire previous turn needs to be re-done by the signer/speaker of the trouble source. On the “strongest” end of the continuum are “understanding-check” formats such as repetition of part or all of a previous turn that invite confirmation that what one just heard or understood was correct. Our proposal for expansion of the continuum is illustrated in Figure 14.

We do not want to imply that the off-record OIR function is the only function of the general behavior of holding the body still while looking at one's interlocutor. The findings of our study apply exclusively to the function of this practice in a specific position in a conversational sequence: i.e., just after a person has been asked a question. Further research is needed to investigate other functions that this behavior may have in other defined types of context, both in LSA, and cross-linguistically.

Finally, our data have come from a sign language, and so one might ask: Is this practice exclusive to sign language? It may not be surprising that we have noticed the “freeze-look” phenomenon in sign language conversation, given that visual behavior is obviously the exclusive focus of attention in this type of language. But users of spoken languages also have a rich set of visual resources at their disposal (McNeill, 1992; Kendon, 2004; Goldin-Meadow, 2005; Enfield, 2009). The “freeze-look” behavior can in principle be produced by anybody in a face-to-face setting, and so we may ask whether it is also used for other-initiation of repair in spoken languages. Only further research will tell, but we see no reason to think it would not be used in this way universally. At least this is a hypothesis to be tested. If the “freeze-look” turns out to be systematically used in spoken language interaction as well, then this study will have made a contribution not only to research on sign languages and on practices for other-initiation of repair in conversation, but it will have taken insights from research on sign language as pointers to an underexplored realm of possibility in spoken language: the systematic use of visible bodily behavior as part of the system of language.

### Conflict of interest statement

The authors declare that the research was conducted in the absence of any commercial or financial relationships that could be construed as a potential conflict of interest.

## Footnotes

^1^Available online at: http://www.censo2010.indec.gov.ar/resultadosdefinitivos_totalpais.asp.

^2^We classify native signers as proficient individuals that have been exposed to a sign language, in this case LSA, from birth or infancy from their caregivers.

^3^The data were transcribed in written Spanish because LSA does not have a written system for this purpose. Most of the signers are bilingual in spoken Spanish and LSA. They use Argentinian Spanish in everyday life for fingerspelling, mouthing, speaking, writing and reading.

^4^They include eyebrow actions (raised and together), wrinkled nose, lips puckered forward, open-mouth gestures (resembling “*Huh?*” in spoken language), head movements (up, down, side, backwards), and leaning forward (Manrique, in press).
