# Supplementary material for: Suspending the next turn as a form of repair initiation: evidence from Argentine Sign Language
Source: Front Psychol. 2015 Sep 15;6:1326. doi: 10.3389/fpsyg.2015.01326 (PMC4569752; doi:10.3389/fpsyg.2015.01326)
Supplement: Supplementary file 1 [file DataSheet1.PDF]

Appendix: Key to Glosses

CAPITAL LETTERS = sign glosses

Egz = eye gaze

EO = eyes wide opened

ER = eyebrows raised

ET = eyebrows together

F-I-N-G-E-R-S-P-E-L-L-I-N-G = finger spelling is indicated by hyphen between letters

G: gesture = followed by a description of the meaning of the gesture, e.g. G:I-don't-mind

G:MM = mouth closed and corners turned down.

H = hold

(sign x)--------H = approximate duration of hold

h-down = head down

h-tilt = head tilt

h-up = head up

HEY-signer (A/B) = One of the signers use one of the getting attentional strategies to call to another signer.

HYPHENATED-WORDS = represent a single sign and more than one English word

LOC = locative

lowercase letters = mouthing translation

LPF = lips puckered forward

MOD = idioms

mth:(reference) = mouthing reference

NEGATIVE-VERB = glossed with the negation in a post verb position, e.g. KNOW-NOT

nod = head nod

NSP = non-signing position

NW = nose wrinkled

OM = mouth wide open

POSS-1 = possessive 1st person (mine)

PRO1 = PRO: pronoun, 1: first person, 2: second person, 3: third person

PT = pointing

PU = palms up

Q = question

SN (description) = sign name

+ = repetition of a sign

Conventions used from oral transcription in spoken languages based on Jefferson (2004; 2015:xiii-xvi).

[ ] Square brackets mark the start and end of overlapping speech. It has been adapted in LSA extracts to indicate only the start of overlapping sign turns between participants ([) using one bigger bracket for both turns instead of single ones for every turn.

(0.5) Parentheses indicate numbers to measure pauses in seconds. It has been adapted in LSA extracts to indicate the duration of the ‘freeze-look’ response.

((text)) Double parentheses contain additional comments about actions noted in the transcript, including non-verbal actions.

(…) Intervening material.

= ‘Latching’: no gap between participants’ turns.

{ } Curly brackets indicate text added by the transcriber that is not present or has not been produced in the original language. This additional text helps to make the translation in English easier to understand.
